# The Relationship between Serum Amyloid A Level and Cognitive Dysfunction in Patients with Vascular Dementia: Preliminary Findings

**DOI:** 10.1155/2021/6676144

**Published:** 2021-02-15

**Authors:** Min Xu, Xiao-ying He, Pan Huang

**Affiliations:** ^1^Department of Neurology, The Second People's Hospital of Deyang City, No. 340 Minjiang West Road, Deyang, Sichuan 618000, China; ^2^Department of Neurology, The Affiliated Hospital of Southwest Medical University, No. 25 Taiping Street, Luzhou, Sichuan 646000, China; ^3^Department of Neurology, People's Hospital of Deyang City, No. 173 TaiShan North Road, Deyang, Sichuan 618000, China

## Abstract

**Objective:**

This study was aimed at investigating the relationship between serum amyloid A (SAA) levels and cognitive dysfunction in patients with vascular dementia (VAD).

**Methods:**

Using cross-sectional research methods, 146 patients with VAD were selected as the VAD group and 70 normal people were selected as the NC group. Upon admission, the clinical and biochemical characteristics of the two groups of study subjects were collected, and the MMSE scale was used to assess cognitive function. A sandwich enzyme-linked immunosorbent assay was used to detect SAA levels.

**Results:**

There was no significant difference in clinical data and biochemical characteristics in the VAD group (*p* > 0.05). Compared with the VAD group, the NC group has a higher level of education (*p* < 0.05). The SAA level of the VAD group was higher than that of the NC group, and there was a significant difference (*p* < 0.05). Spearman correlation analysis showed that SAA and MMSE in the VAD group were negatively correlated. Further multiple regression analysis showed that the serum amyloid A level is an independent risk factor for cognitive dysfunction in VAD patients.

**Conclusion:**

The level of SAA in VAD patients is significantly increased, which can be used as a potential peripheral blood marker to predict cognitive impairment in VAD patients.

## 1. Introduction

Vascular dementia (VAD) refers to a syndrome with a clinical stroke or subclinical cerebrovascular injury that affects at least one cognitive dysfunction. The disease is a heterogeneous brain disease, accounting for more than 20% of dementia, and the prevalence of VAD is 1.0%-8.8% [1]. Studies have speculated that by 2030, VAD patients will reach 66 million, and by 2050, it will reach 120 million [2, 3]. However, in the face of such a large patient population, there is no specific treatment plan for the disease so far, and there is no specific and sensitive molecular marker [4]. Unlike Alzheimer's disease, VAD is potentially preventable. Finding specific markers that can be detected and diagnosed early is conducive to early intervention, delaying the progression of the disease, and reducing the social burden caused by cerebrovascular disease.

SAA is known for its role in secondary amyloidosis. It was isolated and identified from serum in 1976 and is a highly conserved member of the acute phase protein family [5]. The SAA-encoded protein is located in the p15.1 region of chromosome 11, with a size of 150 kb. Human SAA is synthesized in the liver by the cytokines IL-1*β*, IL-6, and tumor necrosis factor-*α* (TNF-*α*) [6]. SAA is the precursor substance of tissue amyloid A and one of the acute phase response proteins. After the body is infected, wounded, or inflamed, the level of SAA rises rapidly [7, 8]. In recent years, with the in-depth study of SAA's gene regulation, protein structure, and biological function, it has been discovered that SAA is also involved in the pathogenesis of many chronic diseases, but the relationship between SAA and VAD is lacking. The main purpose of this study is to detect SAA levels and further clarify whether SAA can be used as a potential biomarker for the prevention and treatment of VAD.

## 2. Materials and Methods

### 2.1. Research Object

We followed the methods of Dr. Juan Li et al. [9]. A total of 146 VAD patients admitted to our hospital from February 2015 to February 2020 were selected as the VAD group, including 70 females and 76 males, aged 40-75 years, with an average of 69.75 ± 7.23 years old. In addition, 70 normal people were selected as the NC group, including 32 females and 38 males, aged 41-78 years old, with an average of 70.31 ± 6.74 years old. The diagnosis of VAD refers to the diagnostic criteria established by the National Institute of Neurological Diseases and Stroke (NINDS-AIREN) and the Diagnostic and Statistical Manual of Mental Disorders (DSM-V) and is determined by two neurologists as the diagnosis of VAD [10, 11]. All VAD patients are diagnosed for the first time and have not been treated. Exclusion criteria are as follows: (1) suffering from other types of dementia; (2) suffering from inflammatory and infectious diseases; (3) suffering from immunological diseases or receiving immunosuppressive treatment; (4) suffering from mental illness; (5) dementia caused by acute cerebrovascular disease or infectious disease; (6) suffering from malignant tumor; (7) operation history or other severe trauma within 3 months; (8) suffering from severe heart, liver, or kidney insufficiency; and (9) used drugs to treat dementia. This study complied with the “Declaration of Helsinki.” We obtained the written consent of all subjects and the approval of the Ethics Committee of Deyang People's Hospital.

### 2.2. Clinical and Biochemical Characteristics

**Figure 1:**
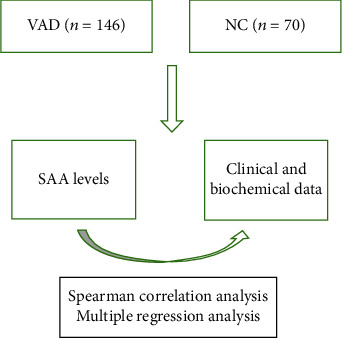
Flow chart.

When admitted to the hospital, the investigators collected the clinical and biochemical characteristics of all patients, including years of education, smoking, drinking, high blood pressure (HBP), diabetes (DM), hyperlipidemia (HLP), body mass index (BMI), systolic blood pressure (SBP), diastolic blood pressure (DBP), total cholesterol (TC), triglycerides (TG), high density lipoprotein cholesterol (HDL-C), low density lipoprotein cholesterol (LDL-C), serum creatinine (Scr), and aspartate aminotransferase/alanine aminotransferase (AST/ALT) (Figure 1).

### 2.3. SAA Determination

In the morning, 5 ml of fasting peripheral venous blood sample was taken from the patient and placed at room temperature to allow it to coagulate naturally for 10-20 minutes. Then, the coagulated blood sample was centrifuged for 30 minutes at 3000 rpm. After centrifugation, the upper serum was carefully separated, collected, and stored in a -80°C ultralow temperature refrigerator. SAA was measured from the stored serum using Invitrogen (Waltham, MA, USA) Sandwich ELISA kit according to the manufacturer's instructions.

### 2.4. Cognitive Function Assessment

The Chinese version of the MMSE scale (Chinese version) was used to evaluate the cognitive function of all patients [12]. The MMSE scale includes six cognitive domains: orientation (time, place), timely memory, delayed memory, attention and calculation, language ability, and visual space perception. The total score of the scale ranges from 0 to 30 points. 24-30 is divided into normal, and <24 is divided into cognitive dysfunction.

### 2.5. Statistical Analysis

The concentration of SAA showed a normal distribution. In this study, categorical variables are recorded by numbers (percentage, %), while continuous variables are recorded by mean ± standard deviation (mean ± SD). The *t*-test was applied for the comparison of continuous variables, and the chi-square test was applied for the comparison of categorical variables. Spearman's correlation analysis is used to assess the binary correlation. Multivariate regression analysis was applied to assess the predictive value of clinical and biochemical characteristics on the cognitive function in patients with VAD. The SPSS17.0 statistical software (SPSS Inc., IL, USA) was used in the study, and a *p* value of 0.05 was considered significant.

## 3. Result

### 3.1. Clinical and Biochemical Characteristics

**Figure 2:**
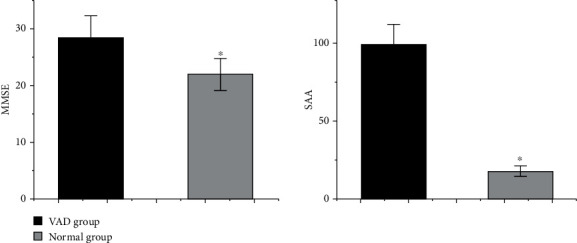
Comparison of MMSE and SAA between VAD group and NC group ^∗^*p* < 0.05.

The study included a total of 216 patients in the Department of Neurology of our hospital from February 2015 to February 2020, including 146 VAD patients and 70 normal controls. The clinical and biochemical characteristics of all subjects are shown in Table 1. In age, gender, smoke and drinking habits, chronic medical history (HBP, DM, and HLP), and biochemical characteristics (BMI, SBP, DBP,TC, TG, HDL-C, LDL-C, BUA, Scr, and AST/ALT), no significant difference was found between the VAD group and the NC group (*p* > 0.05). However, as shown in Figure 2, there are significant differences between the two groups in terms of years of education, MMSE scores, and SAA levels (*p* < 0.05).

### 3.2. Spearman Correlation Analysis

**Figure 3:**
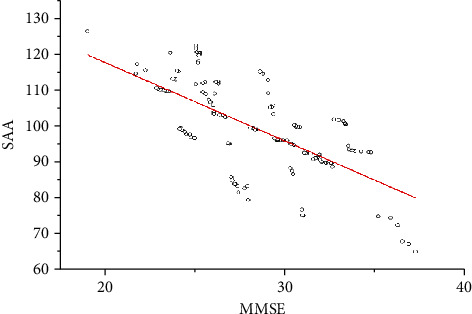
Correlation analysis of MMSE score and various parameters in VAD patients.

Spearman correlation analysis was used to analyze the correlation between clinical and biochemical characteristics and the MMSE score of VAD patients. The results are shown in Table 2 and Figure 3. Spearman correlation analysis showed that the SAA level of VAD patients was negatively correlated with the MMSE score, and the correlation coefficient was significant (*r* = −0.424, *p* < 0.05). However, in our current study, SAA levels are not significantly correlated with the demographic characteristics of VAD patients such as age, gender, education level, smoking habit, drinking habit, HLP, HBP, DM, SBP, DBP, BMI, TC, TG, HDL-C, LDL-C, Scr, BUA, and AST/ALT (*p* > 0.05).

### 3.3. Multiple Regression Analysis

The results of multiple regression analysis of the MMSE score and SAA level of VAD patients are shown in Table 3. The results show that the SAA level can be used as an independent predictive risk factor for cognitive decline in VAD patients. After adjusting for age, gender, years of education, smoking habit, drinking habit, SBP, DBP, TC, TG, HDL-C, LDL-C, FBG, and other clinical and biochemical characteristics, SAA levels are still important for the independent value of VAD cognitive function significance (*β* = 0.427, *p* = 0.024).

## 4. Discussion

The current diagnosis of VAD must meet the following conditions: (1) there must be clinical symptoms of dementia, (2) there must be sufficient evidence of cerebrovascular disease at the same time (including evidence of medical history, physical examination, and radiographic imaging), and (3) the two must be related to each other. In the early 1990s, the diagnostic criteria for VAD were mainly based on AD, emphasizing irreversible cognitive dysfunction and impaired ability of daily living [13]. However, as research progresses, the definition of VAD is considered to be limited because it does not take into account the more common cognitive impairments associated with cerebrovascular diseases, such as executive dysfunction and psychomotor retardation. Therefore, vascular cognitive impairment (VCI) is introduced; VCI refers to a syndrome with a clinical stroke or subclinical cerebrovascular injury that affects at least one cognitive dysfunction, and VAD is the most serious form of VCI [14]. The main purpose of introducing VCI is to better reflect all cognitive changes caused by vascular factors. It is hoped that the vascular factors of cognitive impairment can be identified early and the vascular risk factors can be controlled to slow down the progression of the disease.

This study investigated the relationship between cognitive function and SAA levels in VAD patients and found that compared with normal controls, VAD patients had higher SAA levels and lower MMSE scores. At the same time, it was also found that the SAA level and MMSE score were significantly negatively correlated in VAD patients, but this correlation was not affected by interfering factors such as age, gender, education level, SBP, DBP, BMI, TG, TC, HDL-C, and LDL-C impact. To our knowledge, this is the first time that SAA is an independent risk factor for cognitive impairment in VAD patients. The pathogenesis of VAD is not clear. Vascular mechanisms, decreased cerebral perfusion, small vessel disease, microinfarction, and microhemorrhage are all related to it. Therefore, the factors that can affect the above links may cause the occurrence of VAD [15–18]. The SAA coding genes include SAA1, SAA2, SAA3, and SAA4, mainly SAA1 and SAA2; both genes are 15-20 kb in size, and their promoter region contains nuclear factor-*κ*B (NF-*κ*B) and interleukin-6 (IL-6). Transcription factor recognition sequence, which is the binding site of RAS activator, can be activated by interleukin-1*β* (IL-1*β*), IL-6, and tumor necrosis factor (TNF). From the two coding genes, it can be speculated that the biological function of SAA is related to inflammation. Studies have shown that when the concentration of SAA is as low as 10 mg/l, it has the ability to induce chemokines and chemotactic leukocyte migration. When the concentration of SAA is greater than 10-60 mg/l, it can stimulate endothelial cell proliferation, adhesion, invasion, and formation of a capillary-like structure that stimulates the formation of new blood vessels in the body [19, 20]. In addition, SAA also regulates the outflow or inflow of cholesterol from cells [21]. Current research shows that SAA is related to various pathophysiological processes of atherosclerosis. SAA can stimulate the proliferation and migration of smooth muscle cells, chemoattract neutrophils and monocytes, promote local inflammation, and induce endothelial dysfunction [22]. SAA also increases the synthesis of proteoglycans in vitro and in vivo by inducing transforming growth factor-*β* (TGF-*β*), leading to increased LDL retention in atherosclerotic lesions [23]. In addition, SAA stimulates the formation of foam cells by inducing the endothelial receptors of oxidized low-density lipoproteins and produces a large amount of lipid peroxides. It can also compete with apolipoprotein A1 to bind to HDL, reducing cholesterol transport to the liver and causing lipid deposition [24]. In addition to being related to atherosclerosis, SAA is also related to obesity and type 2 diabetes. There is a large amount of SAA-mRNA expression in liver fat cells of obese patients, and its expression is related to BMI [25]. In type 2 diabetes, the level of SAA is significantly higher than normal, which is mainly related to the fact that SAA can cause insulin resistance, affect the reverse transport of cholesterol, and stimulate mononuclear macrophages to damage vascular endothelium [26–30]. As we all know, atherosclerosis, obesity, and type 2 diabetes are all risk factors for VAD.

Our study found the differential expression of SSA levels in VAD patients for the first time and confirmed that SAA levels are negatively correlated with the cognitive function of VAD patients, which has important clinical potential application value in VAD. But our research also has some limitations. First, the current study is a single-center study with a small sample. Secondly, we did not monitor SAA levels longitudinally nor did we dynamically follow-up patients' cognitive function and prognosis. Third, because the gold standard for diagnosing VAD is biopsy, our diagnosis of VAD may not be accurate enough, and VAD patients may also be accompanied by other types of cognitive impairment. Therefore, larger samples of clinical and basic research are needed for further verification.

## Figures and Tables

**Table 1:** Clinical and biochemical characteristics of all subjects.

Item	VAD group (*n* = 146)	Normal group (*n* = 70)	*p*
Age (years)	69.75 ± 7.23	70.31 ± 6.74	>0.05
Sex (M/F)	76/70	38/32	>0.05
Smoke (*n*, %)	34 (23.28%)	17 (24.28%)	>0.05
Hypertension (*n*,%)	51 (19.02%)	15 (18.75%)	>0.05
DM (*n*,%)	25 (17.12%)	11 (15.71%)	>0.05
Drinking habit (*n*,%)	49 (33.75%)	22 (31.43%)	>0.05
BUA (*μ*mol/l)	353.85 ± 34.23	358.21 ± 35.36	>0.05
TC (mmol/l)	4.79 ± 0.38	4.82 ± 0.41	>0.05
TG (mmol/l)	1.25 ± 0.22	1.28 ± 0.26	>0.05
HDL-C (mmol/l)	0.92 ± 0.21	0.94 ± 0.22	>0.05
LDL-C (mmol/l)	2.91 ± 0.42	2.88 ± 0.43	>0.05
AST/ALT	0.73 ± 0.21	0.74 ± 0.22	>0.05
BMI (kg/m^2^)	23.54 ± 1.28	23.74 ± 1.25	>0.05
Scr (*μ*mol/l)	53.25 ± 6.47	52.87 ± 6.35	>0.05
SBP (mmHg)	135.62 ± 15.36	134.82 ± 15.13	>0.05
DBP (mmHg)	88.46 ± 12.85	87.68 ± 13.36	>0.05
HLP (*n*, %)	30 (20.54%)	14 (20.00%)	>0.05
Education (years)	10.28 ± 2.21	14.63 ± 3.12	<0.05
MMSE (points)	28.53 ± 3.73	21.93 ± 2.78	<0.05
SAA (mg/l)	99.49 ± 12.47	17.97 ± 3.28	<0.05

NC: normal controls; VAD: vascular dementia; SAA: serum amyloid A; HBP: high blood pressure; SBP: systolic blood pressure; DBP: diastolic blood pressure; HLP: hyperlipidemia; TC: total cholesterol; TG: triglycerides; HDL-C: high-density lipoprotein cholesterol; LDL-C: low-density lipoprotein cholesterol; DM: diabetes mellitus; MMSE: Mini-Mental State Examination; Scr: serum creatinine; BUA: blood urea nitrogen.

**Table 2:** Correlation analysis of MMSE score and various parameters in VAD patients.

	SAA	
	*r*	*p*
MMSE	-0.67	<0.05

**Table 3:** Relationship between MMSE score and various parameters in VAD.

	Regression coefficient	*p*	95% CI
Age (years)	0.139	0.176	0.724-1.205
Sex (M/F)	0.256	0.195	0.785-1.189
SBP (mmHg)	0.384	0.433	0.782-1.089
DBP (mmHg)	0.376	0.335	0.551-1.231
BUA (*μ*mol/l)	0.213	0.186	0.736-1.154
TC (mmol/l)	0.271	0.178	0.826-1.214
TG (mmol/l)	0.312	0.569	0.482-1.332
HDL-C (mmol/l)	0.406	0.226	0.779-1.165
LDL-C (mmol/l)	0.263	0.185	0.825-1.127
AST/ALT	0.446	0.202	0.694-1.098
BMI (kg/m^2^)	0.341	0.527	0.476-1.128
Scr (*μ*mol/l)	0.336	0.428	0.628-1.257
Education (years)	0.224	0.076	0.652-1.183
SAA (mg/l)	0.427	0.024	1.635-2.854

## Data Availability

All data, models, and codes generated or used during the study appear in the submitted article.
